# Data for a population based cohort study on abnormal findings of electrocardiograms (ECG), recorded during follow-up periodic examinations, and their association with long-term cardiovascular morbidity and all-cause mortality

**DOI:** 10.1016/j.dib.2019.104474

**Published:** 2019-09-05

**Authors:** Adam Goldman, Hanoch Hod, Angela Chetrit, Rachel Dankner

**Affiliations:** a Department of Epidemiology and Preventive Medicine, School of Public Health, Sackler School of Medicine, Tel Aviv University, Israel; b Leviev Heart Center, Sheba Medical Center, Ramat Gan, Israel; c Unit for Cardiovascular Epidemiology, The Gertner Institute for Epidemiology and Health Policy Research, Ramat Gan, Israel

**Keywords:** Electrocardiogram (ECG), Cardiovascular diseases, Cumulative incidence, Risk factors, Risk prediction, Survival analysis, Screening

## Abstract

In this Data in Brief article, we provide data of the cohort and statistical methods of the research- “Incidental abnormal ECG findings and long-term cardiovascular morbidity and all-cause mortality: a population based prospective study” (Goldman et al., 2019). Extended description of statistical analysis as well as data of cohort baseline characteristics and baseline ECG incidental abnormal findings of 2601 Israeli men and women without known cardiovascular disease (CVD) is presented. The cohort is part of the Israel study of Glucose Intolerance, Obesity and Hypertension (GOH) (Dankner et al., 2007). Furthermore, we provide the data on the performance assessment of the 23 - year CVD-risk and the 31- year all-cause mortality prediction models, which includes Receiver Operating Characteristic (ROC) curves, reclassification-based measures and calibration curve.

**Table:** Specifications Table

Subject	Cardiology and Cardiovascular Medicine
Specific subject area	ECG testing as a primary prevention screening tool in adults without known CVD for early detection of CVD risk and all-cause mortality
Type of data	Tables Graph Figure
How data were acquired	Questionnaires, interviews, physical examination (including anthropometric measurements), laboratory blood tests and ECG recording, performed at regional medical centres or at the homes of the cohort members.
Data format	Analysed Filtered
Parameters for data collection	CVD incidence was determined according to self-reported past myocardial infarction (MI), cerebrovascular accident, peripheral artery disease (PAD) or “other cardiovascular disease” or phase 3 ECG findings of "past MI" or "evidence of myocardial ischemia". All-cause mortality and date of death were recorded from the Israel population registry (May 2017).
Description of data collection	Prospective cohort of 2769 adult men and women randomly selected from the Israel population registry. They were invited to regional clinics during baseline (1979–1984) and during active follow-up (1999–2008) and the data parameters were collected. Several individuals were visited at their homes during the active follow-up since they were too old or had difficulties to travel to the regional clinic.
Data source location	Institution: The Gertner Institute for Epidemiology and Health Policy Research City/Town/Region: Ramat Gan Country: Israel
Data accessibility	With the article
Related research article	Author's name: Adam Goldman, Hanoch Hod, Angela Chetrit, Rachel Dankner Title: Incidental abnormal ECG findings and long-term cardiovascular morbidity and all-cause mortality: a population based prospective study Journal: International Journal of Cardiology DOI: 10.1016/j.ijcard.2019.08.015

**Table:** 

**Value of the data** • These data are important for understanding and interpretation of the potential benefits of the ECG as a screening tool as described in our study [1]. • Clinicians and researchers working in the fields of CVD and diabetes primary prevention, CVD risk prediction and individual's CVD risk stratification. • The full description of the methods, results and prediction models performance measures provide deeper insights regarding CVD risk factors and CVD primary prevention. • These data provide a unique opportunity to follow a high validity data of a representative cohort of healthy women and men over 4 decades for CVD prognostic factors, including baseline ECG findings.

## 1 Data

In this Data in Brief article, we provide the baseline characteristics of the total glucose intolerance, obesity and hypertension (GOH) Israel cohort [2] and Phase-3 CVD incidence for the active follow-up subsample (Table 1). We describe the incidental ECG abnormalities frequencies of the cohort at baseline (Table 2) and summarize the CVD and all-cause mortality according to normal vs. abnormal ECG status (Table 3). The statistical methods for assesing the performance measures of the CVD and all-cause mortality risk prediction models are detailed in 2.1, followed by a summary of these measures (Table 4). The full data of the Net Reclassification Improvement (NRI) following the addition of ECG incidental findings to CVD risk prediction models is also presented (Table 5).

**Table 1:** Baseline characteristics of the total glucose intolerance, obesity and hypertension (GOH) Israel cohort and Phase 3 CVD incidence active follow-up subsample.

	Total cohort (N = 2601) N (%)	CVD follow-up group (N = 930) N (%)	P. value
Sex			
Male	1267 (48.7)	465 (50.0)	0.45
Female	1334 (51.3)	465 (50.0)	
Age			
Years (Mean ± SD)	52.6 ± 8.1	49.0 ± 6.9	<0.001
Year of birth			
1912–1921	763 (29.3)	113 (11.7)	<0.001
1922–1931	963 (37.0)	362 (37.5)	0.769
1932–1941	875 (33.6)	491 (50.8)	<0.001
Origin			
Yemen	648 (24.9)	200 (21.5)	0.037
Middle-East/Asia	652 (25.1)	255 (27.4)	0.166
North Africa	528 (20.3)	156 (16.8)	0.020
Europe/America	773 (29.7)	319 (34.3)	0.009
Smoking			
Never	1573 (60.5)	577 (62.0)	0.342
Former smoker	166 (6.4)	62 (6.7)	
Current smoker	860 (33.1)	291 (31.3)	
BMI (Kg/M^2^)			
Mean (±SD)	26.2 ± 4.3	25.7 ± 3.7	<0.001
Normal	1087 (42.3)	282 (30.6)	<0.001
Overweight	1060 (41.3)	431 (46.7)	0.005
Obese	421 (16.4)	210 (22.8)	<0.001
Blood pressure (mmHg)			
Systolic (Mean ± SD)	132.8 ± 22.0	126.3 ± 18.6	<0.001
Diastolic (Mean ± SD)	84.4 ± 11.5	82.8 ± 11.0	<0.001
Normal	728 (28.4)	359 (38.9)	<0.001
Pre-hypertension	880 (34.3)	309 (33.5)	0.675
Hypertension	957 (37.3)	254 (27.5)	<0.001
Total Cholesterol (mg/dL)			
Mean (±SD)	219.8 ± 54.0	217.5 ± 52.8	0.119
Normal	697 (39.4)	303 (40.2)	
Borderline	446 (25.2)	202 (26.8)	0.141
High risk	627 (35.4)	248 (32.9)	
Creatinine (mg/dL)			
Mean (±SD)	0.96 ± 0.3	0.97 ± 0.4	0.763
Blood glucose			
Normoglycemia	933 (36.1)	309 (33.2)	0.132
Pre-diabetes	1294 (50.0)	465 (50.0)	1.000
Diabetes	361 (13.9)	155 (16.7)	0.041

• Blood pressure classification: Normal-systolic BP ≤ 120 and diastolic BP ≤ 80; Prehypertension- 140 > systolic BP ≥ 120 or 90 > diastolic BP ≥ 80; Hypertension - systolic BP ≥ 140 or diastolic BP ≥ 90.

• Total cholesterol classification: Normal- Total cholesterol <200; Borderline- 200 ≤ Total cholesterol <240; High risk ≥240.

• BMI classification: Normal- BMI <25; Overweight- 25 ≤ BMI <30; Obese- BMI ≥30.

• Diabetes defined if any of the following criteria were fulfilled: FPG ≥126 mg/dL (100–125 mg/dL = prediabetes), OGTT ≥200 mg/dL (140–199 mg/dL = prediabetes), self-report of diabetes or treatment with anti-diabetic drugs.

**Table 2:** ECG abnormal findings according to the Minnesota classification [3] and frequencies (n) in the glucose intolerance, obesity and hypertension (GOH) Phase-2 cohort at baseline.

Single chamber pacemaker (0)	Clockwise rotation (20)	Drug effect (8)
Dual chamber pacemaker (0)	Non-specific T wave changes (II, III, AVF) (284)	Atrial fibrillation (8)
Single SVPB (45)	Non-specific ST-segment changes (II, III, AVF) (277)	Atrial flutter (0)
Multiple SVPB (22)	Non-specific T wave changes (I, AVL, V5-V6) (335)	Atrial tachycardia (1)
Single VPB (45)	Non-specific ST-segment changes (I, AVL, V5-V6) (218)	Diastolic overload (0)
Multiple VPB (26)	Non-specific T wave changes (V1-V4) (200)	Complete left BBB (8)
Low voltage (51)	Non-specific ST-segment changes (V1-V4) (84)	Complete right BBB (29)
Mitral P wave (55)	J point elevation (139)	Intermittent right BBB (1)
Pulmonary P wave (36)	Terminal T negativity (3)	Intermittent left BBB (0)
First degree AV block (51)	Tall T waves (32)	Past MI (0)
Short PR (9)	Prolonged QT (23)	Past MI suspicion (108)- elaborate the followings
Left-axis (<-30°) (168)	Left ventricular hypertrophy (159)	Diaphragmatic (62)
Right axis (>90°) (35)	Right ventricular hypertrophy (6)	Anteroseptal (32)
Incomplete right BBB (114)	Myocardial Ischemia (46)- elaborate the followings	Anterolateral (6)
Incomplete left BBB (21)	Diaphragmatic wall (8)	Anterior (0)
Intraventricular conduction delay (QRS>0.11) (188)	Anterior wall (21)	Lateral (3)
V1- RSR′ pattern (32)	Lateral wall (16)	High lateral (4)
WPW (2)	Posterior wall (1)	True posterior (1)
Poor R wave progression (64)	Left ventricular strain (43)	Subendocardial ischemia (0)
Counterclockwise rotation (330)	Persistent ST-segment elevation (0)	Other (471)

• SVBP- Supraventricular premature beats; VPB- Ventricular premature beats; AV block- Atrioventricular block; BBB- Bundle branch block; WPW- Wolff–Parkinson–White; MI- Myocardial infarction.

• More than one finding was recorded for some individuals.

• Individuals with the following findings were excluded: Single chamber pacemaker, dual chamber pacemaker and past MI.

**Table 3:** CVD 23-year cumulative incidence and 31-year all-cause mortality among individuals with normal ECG tests and those with incidental abnormal ECG findings during Phase-2 GOH data collection.

		Total N (%)	ECG test		P value
			Abnormal ECG findings n (%)	Normal ECG n (%)	
CVD incidence	CVD	294 (31.6)	141 (38.5)	153 (27.1)	<0.001
	No- CVD	636 (68.4)	225 (61.5)	411 (72.9)	
All-cause mortality	Dead	1719 (66.1)	910 (75.9)	809 (57.7)	<0.001
	Alive	882 (33.9)	289 (24.1)	593 (42.3)

**Table 4:** Summary of performance measures for models of 23-year CVD-risk and 31-year all-cause mortality risk prediction.

	CVD			All-cause mortality		
	Traditional risk factors (95% CI)	Traditional risk factors + ECG % (95% CI)	p. value	Traditional risk factors (95% CI)	Traditional risk factors + ECG % (95% CI)	p. value
NRI	a	7.4 (1.5–13.3)	0.01	a	0.6 (−1.3–2.6)	0.52
Continuous NRI	a	25.8 (12.0–39.5)	<0.01	a	41.0 (33.1–48.9)	<0.01
IDI	a	0.63 (0.08–1.17)	0.02	a	0.21 (0.04–0.39)	0.02
C-index	0.656 (0.619–0.694)	0.666 (0.629–0.703)	0.14	0.752 (0.751–0.753)	0.753 (0.752–0.754)	b

CVD = cardiovascular disease, NRI = Net Reclassification Index, IDI = Integrated Discrimination Index.

a Net reclassification improvement is calculated for a model with the addition of ECG findings as compared to a model with traditional risk factors only.

b Comparison of Harrel's C indices for Cox models has unclear reliability [8], thus we calculated 95%CI by bootstrapping (200 repetitions) method and demonstrated a statistically insignificant improvement by confidence intervals overlap.

**Table 5:** Predicted 23-year CVD risk probabilities of 916 seemingly healthy men and women by a multivariable model^a^, with and without ECG findings.

Model without ECG	Model with ECG			Total	Correctly reclassified
Predicted CVD risk^b^	Low <20%	Intermediate 20 - <30%	High ≥30%		
**Participants who experienced a CVD event** n (%)					
<20%	18 (6.2)	7 (2.4)	0 (0.0)	25	
20 - < 30%	8 (2.8)	52 (18.0)	17 (5.9)	77	
≥30%	0 (0.0)	14 (4.8)	173 (59.9)	187	
Total	26	73	190	289	0.69%
**Participants who did not experience a CVD event** n (%)					
<20%	115 (18.3)	22 (3.5)	0 (0.0)	137	
20 - < 30%	42 (6.7)	135 (21.5)	29 (4.6)	206	
≥30%	0 (0.0)	51 (8.1)	233 (37.2)	284	
Total	157	208	262	627	6.7%

Abbreviations: CVD-cardiovascular disease; ECG- Electrocardiogram.

Net Reclassification Improvement (NRI): Overall - 7.39% (95% CI, 1.48%–13.3%, p = 0.014) non-events correctly reclassified (nonevent NRI) - 6.70% events correctly reclassified (events NRI) - 0.69%. Continuous NRI = 25.75% (12.01%–39.50%, p < 0.001), Identification Discrimination Improvement (IDI) = 0.63% (p = 0.024).

a The model is adjusted for: age, sex, origin, BMI, blood pressure, diabetes and smoking status (Model 2).

b Levels of risk are based on ACC/AHA ASCVD Risk thresholds [6] with adjustment to the increased duration of follow-up, similar to Pencina et al. approach [7].

**Fig. 1:**
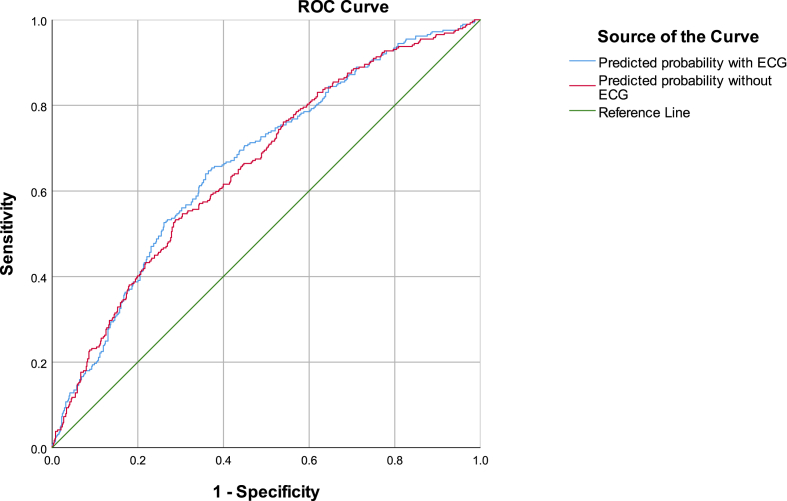
ROC curves of CVD prediction models comprising traditional CVD risk factors^1^, including (blue line) and not including (red line) ECG testing.

**Fig. 2:**
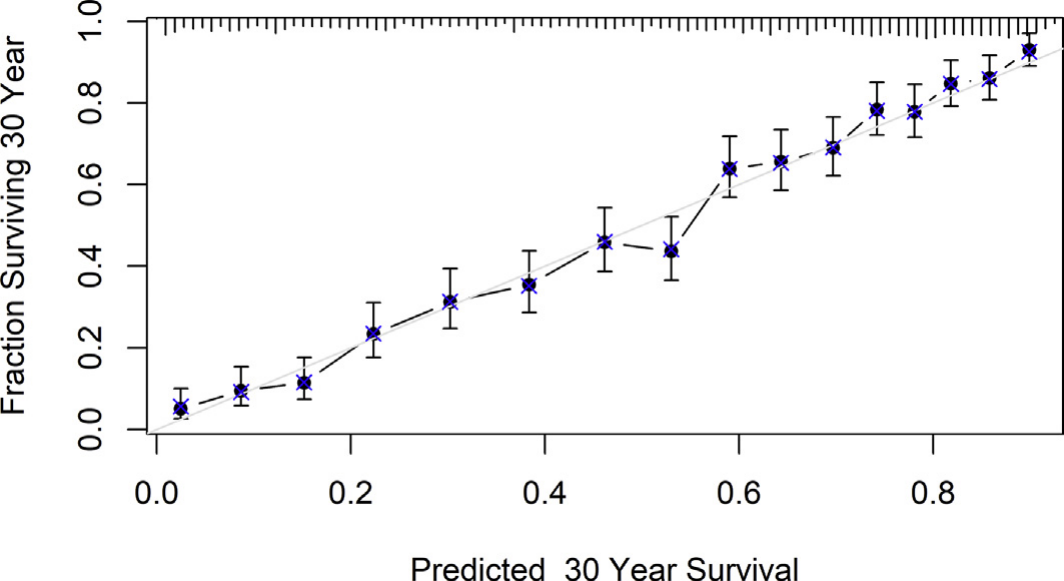
All-cause mortality risk prediction Cox regression model calibration curve.

Fig. 1 shows the ROC curves of CVD risk prediction with vs. without ECG incidental findings. Fig. 2 present the All-cause mortality risk prediction Cox model calibration curve.

## 2 Experimental design, materials, and methods

### 2.1 Assessment of performance measures for CVD and all-cause mortality risk prediction models - statistical methods

To evaluate discrimination improvement, we compared the C-index of the prediction model with traditional CVD risk factors and a model with additional ECG findings. The C-index for the CVD prediction model by logistic regression was calculated by the area under the receiver operating characteristic curve, whereas the C-index for all-cause mortality prediction was calculated by C-index adaption for Cox proportional hazard regression, as proposed by Harrell et al. [4], with the confidence interval calculated by bootstrap resampling with 200 repetitions. We assessed net reclassification improvement (NRI) when incidental ECG findings are added to traditional CVD risk factors at individual risk stratification. The NRI was estimated as described by Pencina et al. [5]:

$$NRI=\left[\frac{\left(\text{number}\;\text{of}\;\text{events}\;\text{reclassified}\;\text{higher}-\text{number}\;\text{of}\;\text{events}\;\text{reclassified}\;\text{lower}\right)}{\text{number}\;\text{of}\;\text{events}}-\frac{\left(\text{number}\;\text{of}\;\text{non}-\text{events}\;\text{reclassified}\;\text{higher}-\text{number}\;\text{of}\;\text{non}-\text{events}\;\text{reclassified}\;\text{lower}\right)}{\text{number}\;\text{of}\;\text{non}-\text{events}}\right]$$

For this purpose, we defined cutoffs for the likelihood to reach the outcome of interest, by adjusting the ACC/AHA [6] risk categories (low, intermediate and high risk) to the increased duration of follow-up, from 10% to 20%–20% and 30%, similar to the Framingham study extension method [7]. We estimated the improvement in reclassification also by continuous NRI measure and the integrated discrimination index (IDI), which are not affected by the chosen cutoff values, in contrast to the NRI measure. Continuous NRI relies on the proportion of individuals with outcome correctly assigned a higher probability and individuals without outcome correctly assigned lower probability, by the new model. IDI reflects the average increase in predicted risk among cases plus the analogous average decrease among controls [5].

Calibration curve of 2520 model 2 participants in all-cause death multivariable analysis. Bootstrap resampling with 200 repetitions for 30-year survival prediction.

## Footnotes

1 ROC curves of a logistic regression model with the covariates: sex, age, origin, blood pressure, BMI and smoking status (model 3). AUC of 0.666 (0.629–0.703), including ECG testing (blue line) vs AUC of 0.656 (0.619–0.694), without ECG testing (red line), p = 0.14.
